# The hypoactive corpora cavernosa with degenerative erectile dysfunction: a new syndrome

**DOI:** 10.1186/1471-2490-6-13

**Published:** 2006-05-24

**Authors:** Ahmed Shafik, Ismail Ahmed, Olfat El Sibai, Ali A Shafik

**Affiliations:** 1 Professor and Chairman, Department of Surgery and Experimental Research, Faculty of Medicine, Cairo University, Cairo, Egypt; 2 Lecturer in Surgery, Department of Surgery and Experimental Research, Faculty of Medicine, Cairo University, Cairo, Egypt; 3 Professor and Chairman, Department of Surgery, Faculty of Medicine, Menoufia University, Shebin El-Kom, Egypt; 4 Assistant Professor of Surgery, Department of Surgery and Experimental Research, Faculty of Medicine, Cairo University, Cairo, Egypt

## Abstract

**Background:**

In a group of 22 patients with erectile dysfunction, vasculogenic, neurogenic, endocrinologic or psychogenic investigations failed to find a cause for their erectile dysfunction. The electro-cavernosograms of these patients recorded a diminished activity. We investigated the hypothesis that diminished corpus cavernosum electromyography activity was the cause of erectile dysfunction in these patients.

**Methods:**

The study comprised the above mentioned 22 patients (study group, 43.8 ± 5.9 SD years) and 15 healthy volunteers (control group, 41.8 ± 5.1 SD years). The electro-cavernosograms were recorded in the flaccid, erectile and detumescent phases by 2 electrodes inserted into the corpus cavernosum.

**Results:**

The electro-cavernosogram of the healthy volunteers registered in the flaccid phase regular slow waves and random action potentials. The wave variables declined significantly in the erectile phase (p < 0.01). In the study group, the slow wave variables in the flaccid phase exhibited a significant decrease (p < 0.05) compared to the healthy volunteers, and the rhythm was irregular. Erection did not occur with sildenafil administration or intracavernosal papaverine injection, and penile implant was performed. Biopsy examination showed degenerated muscle fibers, and fragmented collagen and elastic fibers with areas of fibrosis.

**Conclusion:**

A novel concept of the cause of erectile dysfunction was presented. Corpora cavernosa showed degenerative changes on histopathologic examination and exhibited diminished electromyography activity. They did not respond to sildenafil administration or intracavernosal papaverine injection. Penile implants were the only treatment. The condition is given the name 'hypoactive corpus cavernosum'. The cause of corpus cavernosum degenerative changes needs further study.

## Background

Penile erection is produced by relaxation of cavernosus smooth muscles of the penis with a resulting filling of the sinusoids of the corpus cavernosum with blood. Penile erection and detumescence depend on relaxation and contraction of the cavernous smooth muscle [1-5]. The male erectile response is a neurovascular event depending on the complex interaction between neurological and vascular responses [6-8]. Erectile dysfunction is usually multifactorial and has been classified as vasculogenic, neurogenic, endocrinologic or psychogenic. Impaired blood flow to the penis is the most common cause of ED [1-6]. Altered neural function is generally regarded as the second common cause of ED, although its contribution is commonly underestimated as it is difficult to assess [6].

Relaxation of the smooth CC muscles is considered as the key mechanism of erection. Recording of the electric activity of the CC (CC EMG), or electrocavernosography, was introduced by Wagner and Gerstenburg in 1989 [9] and later studied by other investigators [10-16]. Electrocavernosography provides information on the smooth musculature and autonomic innervation of the penis. It has been introduced into the diagnostic evaluation of ED [10-16].

During our study of the cause of erectile dysfunction in 652 patients, we encountered a group of 22 patients in whom all investigations have failed to recognize a cause of their ED. A vasculogenic, neurogenic, endocrinologic or psychogenic cause was not found.

A recent study has demonstrated a condition we termed 'overactive corpus cavernosum' as a cause of ED [15]. In these cases, an elevated electromyographic (EMG) activity of the CC muscle fibers was detected in the flaccid phase. This augmented activity presumably denotes hypertonicity of these fibers and might explain their failure to relax to effect erection.

When we studied the EMG activity of the CC of the above mentioned 22 patients with ED, a diminished activity was recorded. We hypothesized that the diminished electric activity of the CC or the 'hypoactive CC' is the cause of ED in these patients. This hypothesis was investigated in the current study.

## Methods

### Subjects

The study comprised 22 patients (study group, mean age 43.8 ± 5.9 SD years, range 37–52) with diminished CC EMG activity and with ED of a mean duration of 14.4 ± 2.2 SD years (range 10–16), and 15 healthy volunteers (control group, mean age 41.8 ± 5.1 SD years, range 34–50) who matched the patients in age and acted as controls. The volunteers had a normal sexual life and normal erection. All the subjects in the study gave an informed written consent after having been fully informed about the nature of the study, the tests to be done, and their role in the study.

The results of the physical examination including neurologic assessment were unremarkable. The laboratory work which comprised blood count, renal and hepatic function tests, and electrocardiogram recorded normal findings. The investigations for ED were done for the patients and healthy volunteers. These investigations included cavernosometry, cavernosography, penile arteriography, penile biothesiometry, penile Doppler and rigiscan. **Duplex ultrasound scanning was performed in conjunction with a papaverine test **[17]. **The mean arterial diameter increase was 88.2 % (range 82 – 93 %) of the flaccid value, and the mean peak flow velocity was 38.2 cm/s (range 33 – 48) after papaverine injection; these values represent adequate arterial capacity. During the 3 nights of testing the nocturnal penile tumescence (NPT), the greatest registered penile circumference was noted on the recorded data. If direct observation would estimate this circumference as a full erection, then episodes with a circumference of between 81 and 100 % of this greatest circumference would be considered to be a maximal episode **[18]. **All of the studied patients had an average of 4 erectile episodes per night (range 3 – 5) with a mean maximal rigidity of 88.5 ± 3.2 % (range 84 – 92)**. Neurophysiologic studies were performed comprising of bulbocavernosus-evoked potential testing, dorsal nerve conduction velocity and dorsal nerve somatosensory-evoked potential testing. Neurogenic, vasculogenic, endocrinal and psychological causes of ED were excluded as a result of these investigations (table 1).

The study was approved by the Review Board and Ethics Committee of the Cairo University Faculty of Medicine.

### Methods

The electric activity of the CC was recorded at rest (flaccid phase) and during erection and detumescence by means of a concentric electromyographic (EMG) needle electrode (Type 13L49 Disa, Copenhagen, Denmark) measuring 40 mm in length and 0.65 mm in diameter. Two needle electrodes were introduced into the CC: one in the upper and one in the lower third. A ground electrode was applied to the thigh and a strain-gauge respiratory transducer to the thoracic wall. After recording the electric activity, the upper electrode was transferred to the mid third of the CC and the recordings were repeated. The needle electrodes were then transferred to the contralateral CC and the electric activity was recorded.

A standard EMG apparatus (Type MES, Medelec, Woking, UK) was used to amplify and display the recorded potentials. Films of these potentials were taken on a light-sensitive paper (Linagraph type 1895, Kodak, London, UK) from which measurements of the duration of the motor unit action potentials were obtained. The EMG signals were in addition stored on an FM tape recorder (type 7758A, Hewlett-Packard, Waltham, MA) for further analysis as required. All filtered signals were collected and recorded using an online computer with a data acquisition and analysis software (Chart V 4.2, AD instruments, Castle Hill, Sydney, Australia). The acquisition rate was 10 Hz, and the EMG normal band width was 0.1 to 5.0 Hz.

Following the recording of the electric activity in the flaccid phase, erection was induced by administration of sildenafil citrate tablets and audiovisual sexual stimulation. One 50 mg tablet was given orally, and the EMG recording of the CC electric activity was performed as aforementioned after erection had occurred. If no erection had been achieved one hour after administration of 50 mg sildenafil, the EMG recording was discontinued and repeated 2 days later; if after 1 hour from administration of 100 mg of sildenafil and audiovisual sexual stimulation erection had as yet not been obtained, intracavernosus injection of papaverine was used to induce erection [19].

To ensure reproducibility of the results, the recordings were repeated at least twice in the individual subject. The results were analyzed statistically using the Student's *t *test, and values were given as the mean ± standard deviation (SD). Differences assumed significance at p < 0.05.

### Corpus cavernosum biopsy

Penile implant was performed in the 22 patients of the study group who had all consented to do the penile implant. They also gave an informed consent for biopsies to be taken from each CC at operation. The obtained corporeal tissue was stained with hematoxylin and eosin and Mallory's trichrome stain. The healthy volunteers refused to do a CC biopsy for control.

## Results

All the subjects were evaluated and no adverse side effects were encountered during or after the study.

### Electrocavernosogram (ECG) of the healthy volunteers

**Figure 1:**
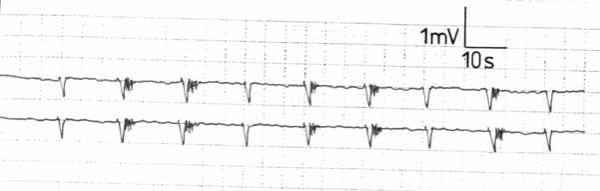
Electrocavernosogram in the flaccid phase of a healthy volunteer showing slow waves with regular rhythm and random action potentials. The recordings from the 2 electrodes were identical.

Slow waves (SWs) were recorded in the flaccid phase from the two electrodes of each individual. The wave was negatively deflected (fig. 1) and had an invariable shape in all the recordings from the same site. The SWs in each individual exhibited the same frequency, amplitude and conduction velocity by the 2 electrodes (fig. 1) regardless of their location in the upper, middle or lower third of the penis. Frequency, amplitude and conduction velocity were constant in each subject. The SW variables in the flaccid phase are shown in table 2. These values were reproducible from the electrodes in the upper, middle or lower third of the penis and were identical from both CCs of the same subject. Bursts of fast activity spikes or action potentials (APs) superimposed or followed the SWs. They presented as negative deflections, and their frequency was inconsistent in each subject (fig. 1). The described SW pattern was identical from the 2 CC of the individual subject.

**Figure 2:**
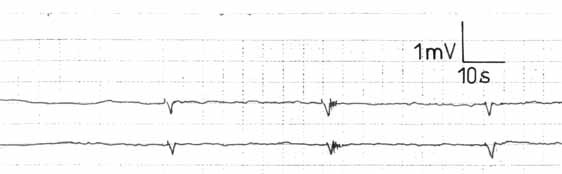
Electrocavernosogram during rigid erection of fig. 1 showing lower frequency, amplitude and conduction velocity of the slow waves than during the flaccid phase. The action potentials were also less frequent. The recordings from the 2 electrodes were identical.

The ECG during rigid penile erection registered significantly diminished CC electric activity (fig. 2, table 3). The frequency, amplitude and conduction velocity showed a significant decrease when compared to the flaccid phase (p < 0.01, p < 0.01, p < 0.01, respectively; table 3). The APs occurred also randomly, but with diminished frequency. These findings were identical from the 2 electrodes of the same subject and from the upper, middle and lower third of the penis, and were reproducible. The recording of the electric activity after detumescence was not significantly different from that of the flaccid phase.

### Electrocavernosogram of the ED group

**Figure 3:**
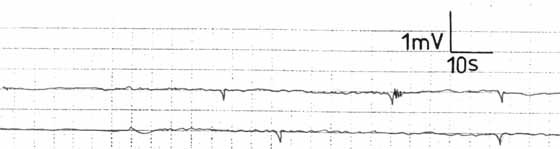
Electrocavernosogram in the flaccid phase of a patient of the ED group showing diminished frequency, amplitude and conduction velocity compared to those of the healthy volunteers. The rhythm was irregular and the recordings from the 2 electrodes of the same subject were not identical.

In the flaccid phase, the SWs were negatively deflected. The frequency, amplitude and velocity of conduction were significantly reduced (p < 0.05, p < 0.05, p < 0.05, respectively) in comparison to the normal controls (fig. 3, table 2). The wave rhythm was irregular, and the recordings from the 2 electrodes of the same individual were not identical (fig. 3). The irregular rhythm was consistent when the examination was repeated in the same subject. The APs occurred rarely and randomly; they were less frequent if compared to those of the healthy volunteers (fig. 3). The SW pattern was also recorded from the opposite CC of the same patient, although the SW variables were not identical.

**Figure 4:**
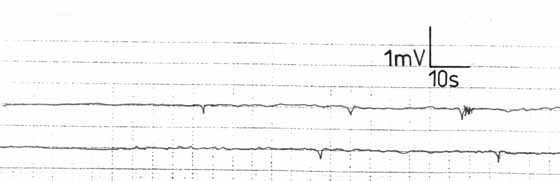
Electrocavernosography in the flaccid phase of the same patient of fig 3 after 100 mg sildenafil administration and audiovisual sexual stimulation showing no response.

**Figure 5:**
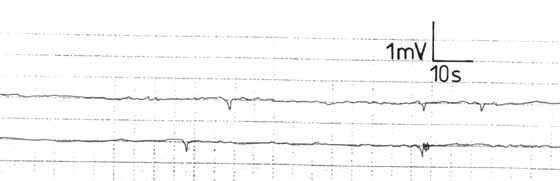
Electrocavernosography in the flaccid phase of the same patient of fig. 3 after intracavernosal papaverine injection showing no response.

Erection could not be initiated in any of the patients after 50 and 100 mg sildenafil administration and audiovisual sexual stimulation (fig. 4), nor did intracavernosal injection of papaverine effect erection (fig. 5). The ECG performed while the patients were under the effect of sildenafil or papaverine did not differ significantly from the ECG prior to treatment; the SW variables are shown in table 3.

### Results of the microscope examination of the CC biopsies

**Figure 6:**
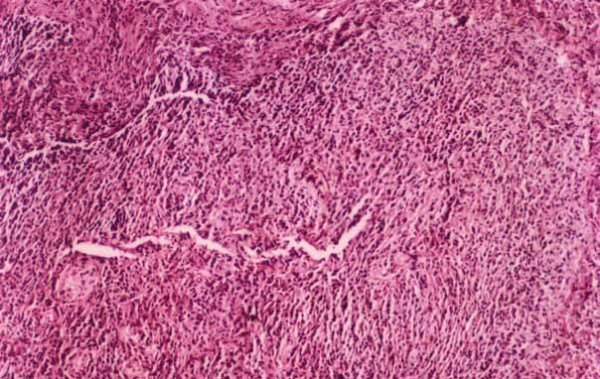
Photomicrograph of a biopsy specimen from the corpora of a patient showing fragmented collagen and elastic fibers and degenerated cavernosal muscle fibers Mallory's trichrome stain X100.

**Figure 7:**
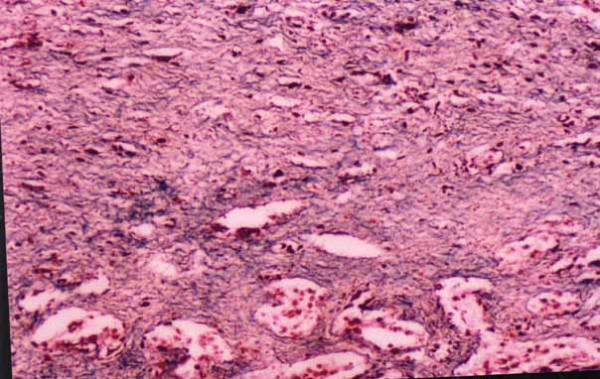
Photomicrograph of a biopsy specimen from the corpora cavernosa of a patient showing obliterated degenerated cavernous tissue, and areas of fibrous tissue Mallory's trichrome stain X100.

The biopsies from the CC of the patients showed fragmented collagen and elastic fibers, degenerated muscle fibers and areas of fibrosis (fig. 6, 7). In some specimens, there were in addition non-specific inflammatory cells and thrombosed vessels. These histopathologic findings were present in the biopsies from both CCs of each individual and in all of the studied patients.

## Discussion

Despite the advances made in diagnosis and treatment of ED, there is still what we call the idiopathic ED of hitherto unknown cause. Classically, ED may be due to neurogenic, vasculogenic, endocrinal or psychogenic factors [1-6]. However, the idiopathic group does not fall under any of these categories. The current communication presents a group of patients with idiopathic ED who did not respond to any form of treatment including sildenafil citrate or papaverine intracavernosal injection. Electromyographic studies of the CCs of these patients revealed a diminished EMG activity compared to the healthy volunteers.

Under normal physiologic conditions, the cavernous smooth muscles are in a state of contraction. Penile erection is mainly effected through cavernous smooth muscle relaxation and the filling of the CC sinusoids with arterial blood. Penile erection and detumescence depend on cavernous smooth muscle relaxation and contraction, respectively. In the current study, the EMG activity of the smooth CC musculature was reduced, denoting presumably a diminished tone. Also there was no response to sildenafil administration or intracavernosal injection of the CC. The cause of these changes in the CC is unknown and needs to be discussed.

The investigative results of the ED patients excluded the possibility of one of the known causes of ED to be related to the condition. The histologic study showed that the smooth musculature of CCs exhibited degenerative changes in the form of fragmented collagen and elastic fibers as well as degenerated muscle fibers. These degenerative changes appear to be responsible for the diminished tone of the cavernous tissue as detected by electrocavernosogram. The tonic contraction of the CC musculature is sympathetic in nature. Investigators have shown that detumescence and cavernosal tissue contraction were sympathetic phenomena that could be mediated by various peripheral pathways which were contained exclusively in the hypogastric nerves [20]. They concluded that impulses are mediated via a sympathetic pathway passing through the inferior mesenteric ganglia and hypogastric nerves to the pelvic plexus. The sympathetic nervous system has a potentially important role in the mediation of penile tumescence as well as in detumescence responses [20]. The cause of the diminished cavernosal muscle tone in the patients of the current study may be due to inhibited sympathetic innervation with secondary degenerative changes in the CC musculature. However, although these patients were neurologically free, yet they might need a specific study of the sympathetic innervation of the CC. Alternatively, the CC degenerative changes may be ischemic resulting from penile ischemia. However, our studies have excluded, as above mentioned, the presence of a vasculogenic factor. Other causes of muscular degenerative changes such as neurologic diseases may be accused, but were excluded by clinical examination and investigative results. We call this condition 'hypoactive CC'. Further studies need to be conducted before the condition can be considered as being primary degenerative.

The treatment of these patients is problematic. The subjects did not respond to sildenafil or intracorporeal injection treatment. This is probably due to failure of the degenerative CC musculature to relax with these treatments. Penile implants appear to be the only treatment for these patients. The cavernous tissue is already degenerated and the implant insertion seems to be an appropriate indication for the treatment of such cases.

## Conclusion

The study presented a novel concept of the cause of ED in 22 patients. The known causes of ED did not exist in these patients. Corpora cavernosa showed degenerative changes on histopathologic examination. They exhibited diminished EMG activity and did not respond to sildenafil administration or intracavernosal papaverine injection. We call this condition 'hypoactive CC'. Penile implants were the only satisfactory treatment. The cause of these degenerative changes need further studies.

## Abbreviations

erectile dysfunction = (ED)

electro-cavernosograms = (ECGs)

corpus cavernosum = (CC)

slow waves = (SWs)

action potentials = (APs)

## Competing interests

The author(s) declare that they have no competing interests.

## Authors' contributions

AS carried out the study design, data collection, statistical analysis, data interpretation and preparation of manuscript. IA participated in data collection and analysis and literature search. OE participated in data collection, statistical analysis and literature search. AAS participated in data collection and preparation of the manuscript. All authors read and approved the final manuscript.

## Pre-publication history

The pre-publication history for this paper can be accessed here:



## Figures and Tables

**Table 1:** Investigations performed in excluding organic erectile dysfunction in the studied patients.

- Bulbocavernosus-evoked potential
- Dorsal nerve conduction velocity
- Dorsal nerve somatosensory-evoked potential
- Penile Doppler
- Rigiscan
- Cavernosometry
- Cavernosography
- Penile arteriography
- Penile biothesiometry
- Serum prolactin, LH, FSH
- Serum testosterone

**Table 2:** The frequency, amplitude, and conduction velocity of the slow waves recorded in the flaccid phase of the healthy volunteers, and ED group^+^.

**Slow waves**						
	**Frequency cycles/min**		**Amplitude mV**		**Conduction velocity cm/s**	

	**Mean**	**Range**	**Mean**	**Range**	**Mean**	**Range**

**Healthy volunteers**	4.2 ± 1.2	3.3–5.9	0.53 ± 0.07	0.42–0.75	4.6 ± 0.7	3.9–5.3
**ED group**	1.4 ± 0.03*	1.2–1.8	0.16 ± 0.02*	0.12–0.20	1.4 ± 0.07*	1.1–1.8

+ values were given as the mean ± SD

* p < 0.05

p p-values were compared to those of the healthy volunteers

**Table 3:** The frequency, amplitude, and conduction velocity of the slow waves recorded one hour after 100 mg of sildenafil administration in the healthy volunteers and ED group^+^.

**Slow waves**						
	**Frequency cycles/min**		**Amplitude mV**		**Conduction velocity cm/s**	

	**Mean**	**Range**	**Mean**	**Range**	**Mean**	**Range**

**Healthy volunteers**	1.6 ± 0.5	1.3–2.1	0.12 ± 0.01	0–0.2	1.6 ± 0.3	1.2–2.1
**ED group**	1.4 ± 0.3	1.1–1.7	0.15 ± 0.02	0.11–0.21	1.3 ± 0.06	1.1–1.6

+ values were given as the mean ± SD
